# A carrier velocity model for electrical detection of gas molecules

**DOI:** 10.3762/bjnano.10.64

**Published:** 2019-03-04

**Authors:** Ali Hosseingholi Pourasl, Sharifah Hafizah Syed Ariffin, Mohammad Taghi Ahmadi, Razali Ismail, Niayesh Gharaei

**Affiliations:** 1 UTM-MIMOS Center of Excellence in Telecommunication Technology, School of Electrical Engineering, Faculty of Engineering, Universiti Teknologi Malaysia, 81310 Skudai, Johor, Malaysia; 2 Nanotechnology Research Center, Nanoelectronic Group, Physics Department, Urmia University, 57147 Urmia, Iran; 3 Computational Nanoelectronic Research Group (CoNE), School of Electrical Engineering, Faculty of Engineering, Universiti Teknologi Malaysia, 81310 Skudai, Johor, Malaysia; 4 School of Computing, Faculty of Engineering, Universiti Teknologi Malaysia, 81310 Skudai, Johor, Malaysia

**Keywords:** armchair graphene nanoribbons, carrier velocity, gas sensor, *I*–*V* characteristics, molecular adsorption

## Abstract

Nanomaterial-based sensors with high sensitivity, fast response and recovery time, large detection range, and high chemical stability are in immense demand for the detection of hazardous gas molecules. Graphene nanoribbons (GNRs) which have exceptional electrical, physical, and chemical properties can fulfil all of these requirements. The detection of gas molecules using gas sensors, particularly in medical diagnostics and safety applications, is receiving particularly high demand. GNRs exhibit remarkable changes in their electrical characteristics when exposed to different gases through molecular adsorption. In this paper, the adsorption effects of the target gas molecules (CO and NO) on the electrical properties of the armchair graphene nanoribbon (AGNR)-based sensor are analytically modelled. Thus, the energy dispersion relation of AGNR is developed considering the molecular adsorption effect using a tight binding (TB) method. The carrier velocity is calculated based on the density of states (DOS) and carrier concentration (*n*) to obtain *I*–*V* characteristics and to monitor its variation in the presence of the gas molecules. Furthermore, the *I*–*V* characteristics and energy band structure of the AGNR sensor are simulated using first principle calculations to investigate the gas adsorption effects on these properties. To ensure the accuracy of the proposed model, the *I*–*V* characteristics of the AGNR sensor that are simulated based both on the proposed model and first principles calculations are compared, and an acceptable agreement is achieved.

## Introduction

The unique electrical, physical, and chemical properties of graphene nanoribbons (GNRs) make them very interesting for use in the future generation of the electronic devices, such as field effect transistors (FETs), diodes, capacitors, memories, and sensors [1–2]. Compared to its counterparts, such as silicon nanowires and carbon nanotubes, GNRs possess high sensitivity, high electron and hole mobility, chemical stability, low noise, and a large surface-to-volume ratio, properties which are highly desired for gas sensor applications. Electrically, GNRs have shown high sensitivity to their surroundings and adsorption or desorption of molecules [3–4]. Thus, many studies have been conducted regarding the synthesis, characterization and implementation of GNRs for the detection of gas molecules and biomolecular species. The importance of the GNR-based electrodes, surface functionalization techniques, and detection approaches have been studied, and their electrical properties have also been investigated [5–9]. In addition, many researchers have experimentally worked on the fabrication of graphene and GNR-based biosensors and gas sensors [10–15]. Most of the previous works are experimental or theoretical simulation studies that investigated graphene, zigzag GNR or their composite material forms. Despite the remarkable advancement in recent years, this area is still in its initial development stage. Some critical issues, such as finding an ideal material and the enhancement of sensing methods based on electronic detection techniques, are receiving increasing attention [16–18]. The experimental detection techniques have some constraints such as low detection range, high cost, and mechanism complexity [19–20]. Theoretical methods and analytical techniques present a proper path forward to overcome the constraints of experimental approaches. The adsorption of gas molecules can modulate different electrical and physical properties of the GNRs, such as density of states (DOS), carrier concentration, carrier velocity, *I–V* characteristics, and energy band structure. On the other hand, the charge transfer between GNRs and the adsorbed target molecule can change the concentration of the carriers and modulate the conductivity of the GNR. In addition, because of the atomic forces and charge transfer, molecular adsorption can lead to modification of the energy band diagram and variation of GNR energy band gap, and conversion of GNR to a n-type or p-type semiconducting material depending on whether it is an acceptor or donor functional molecule [21]. On the other hand, the variation of the energy band gap could convert the GNR properties from metallic to semiconducting, or vice versa. All these phenomena lead to the variation of the velocity of the electrons and change the current–voltage properties of the sensor. These effects are important factors that can be used as sensing parameters in the sensor modelling. In this paper, the fundamentals and working principles of gas sensors are used to develop a new gas sensor model based on the carrier velocity and *I*–*V* characteristics. In addition, a first principle simulation study is employed for the band structure analysis, to calculate the charge transfer, and to evaluate the proposed models. For the sensor structure, an armchair graphene nanoribbon based field effect transistor (AGNR-FET) is used as the sensor platform.

## Modelling and Formalism

**Figure 1:**
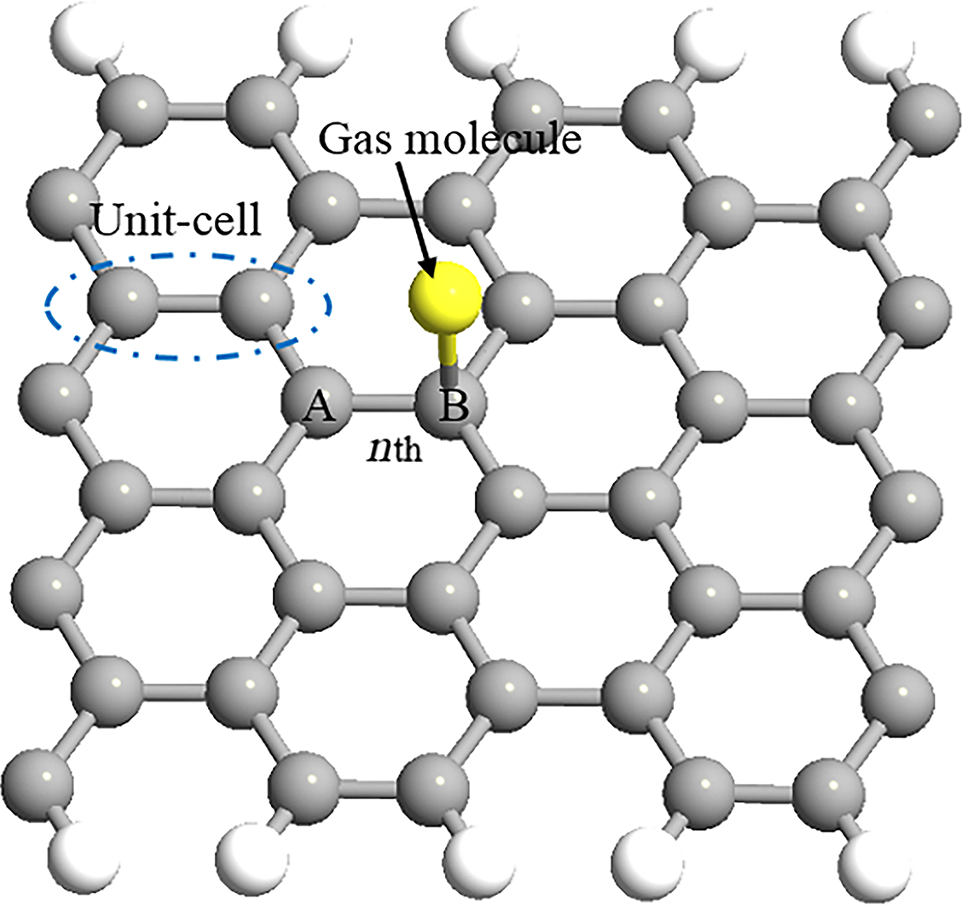
Illustration of the gas molecule adsorption on the armchair graphene nanoribbon (AGNR) unit cell.

In this study, AGNR as a 1D carbon material that contains a pair of atoms in the unit cell is incorporated with the assumption that for each carbon atom there is only one orbital leading to the following Schrödinger equation [22]:

[1]
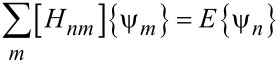


where {ψ*_m_*_ or (_*_n_*_)_} is a column vector indicating the wave function in unit cell *m* (or *n*), *H_nm_* is the Hamiltonian matrix and *E* is the energy. Based on a theory that uses one orbital (the s orbital) per carbon atom, a 2 × 2 matrix describing the conduction and valence band can be introduced. The matrix element between neighbouring carbon atoms as upper diagonal or lower diagonal of the matrix was assumed to be equal to *t*, whereas other elements are assumed to be equal to zero [23–24]. In our model, the adsorption of gas molecules on a second atom (we consider the B atom) of the *n*th unit cell of graphene is assumed, as shown in Figure 1. Here, the adsorption of each gas molecule on a single carbon atom is assumed (i.e., single contact).

In the modelling of the energy band structure of AGNRs considering the molecular adsorption effect, the tight-binding (TB) approximation technique based on the nearest neighbourhood is used for both the AGNR and gas molecules. We consider a 2D nanosheet made up of *N* sites. There are four nearest unit cells around the *n*th unit cell. Using the TB approximation technique, the Schrödinger equation becomes:

[2]
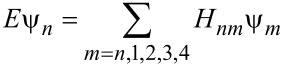


where the Hamiltonian matrixes *H_n,m_* (*m* = *n*, 1, 2, 3, 4) indicate the coupling between the adsorbed molecule and carbon atom, and ψ*_n_* is the vector representing the wave function in the *n*th unit cell. It is noteworthy that only the nearest neighbour interactions in terms of the *n*th unit cell appear in the expression. Therefore, based on the TB model, our Hamiltonian matrix will have 3 × 3 dimensions. For the sake of simplicity, the extended form of Equation 2 is addressed as *h*(*k*). Based on Equation 2, the eigenequation for eigenvalue *E* is written as:

[3]
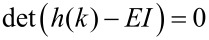


where *I* is the identity matrix. Equation 3 can be solved in the following form:

[4]
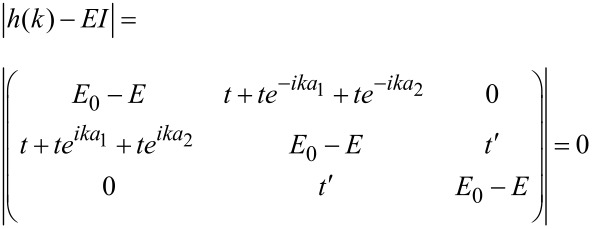


where *a*_1_ and *a*_2_ are the lattice vectors. After calculation of the determinant of the matrix above, and applying the Taylor expansion and the quantum confinement effect [25] due to the finite width, the energy dispersion relation can be obtained for AGNR considering the gas molecules adsorption effect as:

[5]
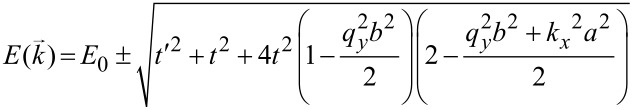


[6]
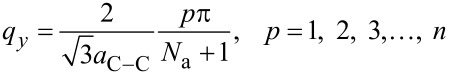


where *t* = 2.7 eV is the carbon–carbon tight-binding overlap energy. The *E*_0_ is described as the on-site energy, *t′* is the overlapping energy between the AGNR and gas molecule (AGNR–gas hopping integral parameter), *a*_C–C_ is the C–C bond length (*a*_C–C_ = 1.42 Å), *b* is lattice parameter and the wave vectors along the *x* and *y* directions are represented by *k_x_* and *k_y_*. In addition, *N*_a_, *p* and *q_y_* are the number of dimmer lines, number of sub-bands, and quantized wave vector, respectively. It is obvious that the quantity of *t′* can be different depending on the geometry and type of gas molecules.

## Results and Discussion

In the modelling of the gas sensor, the velocity of the carriers can be used as one of the key parameters to discover and investigate the molecular adsorption effects on the electrical properties of the sensor, which is our focus in this paper. Furthermore, experimental and theoretical studies have confirmed that the velocity of the electrons is a function of carrier concentration (*n*) and density of states (DOS). The electron’s velocity is directly proportional to the DOS at any instance. The carrier velocity in the AGNR can be obtained by the accumulative velocity of all the carriers over the density of carriers, and is given by [26]:

[7]
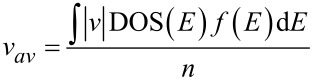


where


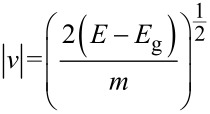


is the magnitude of velocity (*m* is electron mass and *E*_g_ is energy band gap), and


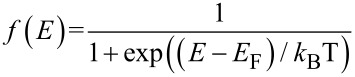


is the Fermi–Dirac distribution function that indicates the probability of occupation of a state at each energy level [27]. The DOS indicates the number of available energy states per energy interval at each energy level that can be occupied by the electrons. By taking the derivative of energy over the wave vector, *k_x_*, the density of states for the AGNR considering the effect of gas molecule adsorption is calculated as:

[8]
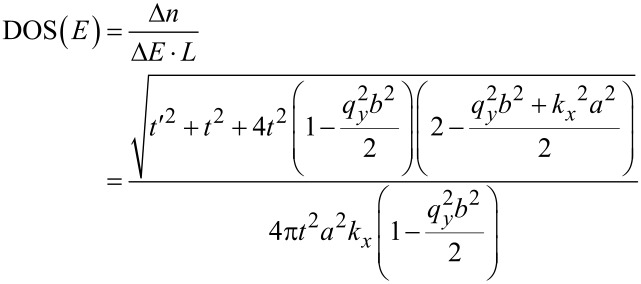


where the wave vector along the *x* direction is obtained as:

[9]
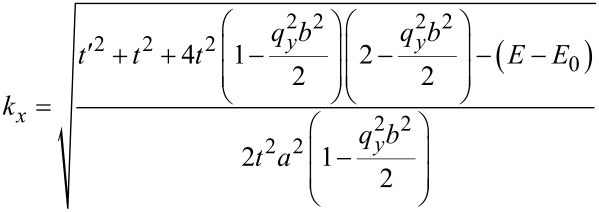


For the bare AGNR, the value of the hopping integral parameter is *t′* = 0 eV. The value of the DOS near the Fermi level would be equal to 0 (eV)^−1^ for the bare AGNR and the width of zero DOS area should be equivalent to the energy band gap value of bare AGNR. This is because there are no electrons hopping from the valence band to the conduction band at the energy gap, as there are no energy states at the band gap.

In addition to the DOS, we first need to calculate the carrier concentration and then we can calculate the carrier velocity. In order to calculate carrier concentration, the density of carriers (electrons) has been extracted to be DOS(*E*) × *f*(*E*) × d*E*. Hence, the concentration of carriers would be calculated by integration of the Fermi distribution function over energy [28]. With *E* = (*E*_g_ – *x*)*k*_B_*T*, normalized Fermi energy η = (*E*_F_ – *E*_g_)/(*k*_B_*T*) and *x* = (*E* – *E*_g_)/(*k*_B_*T*) [29], the carrier concentration including the gas adsorption effect can be formulated for AGNR-FET-based gas sensors.

[10]
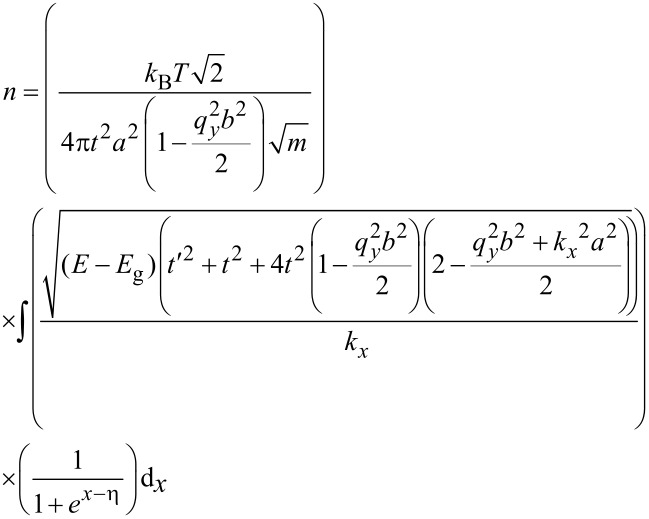


where *k*_B_ is the Boltzmann constant and *T* is the temperature. We now have all the information required to calculate the carrier velocity. Finally, based on Equation 7, the carrier velocity for the AGNR including the molecular adsorption effect can be modeled as:

[11]
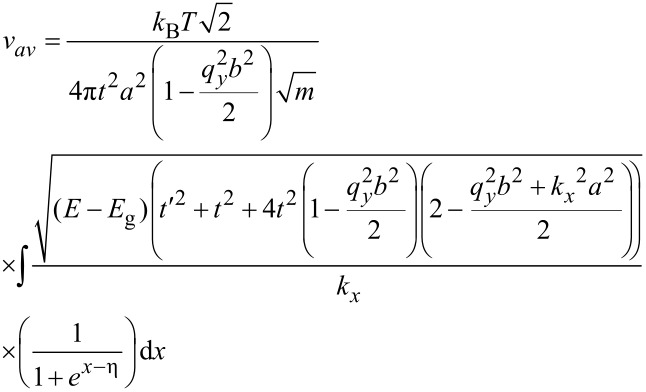


**Figure 2:**
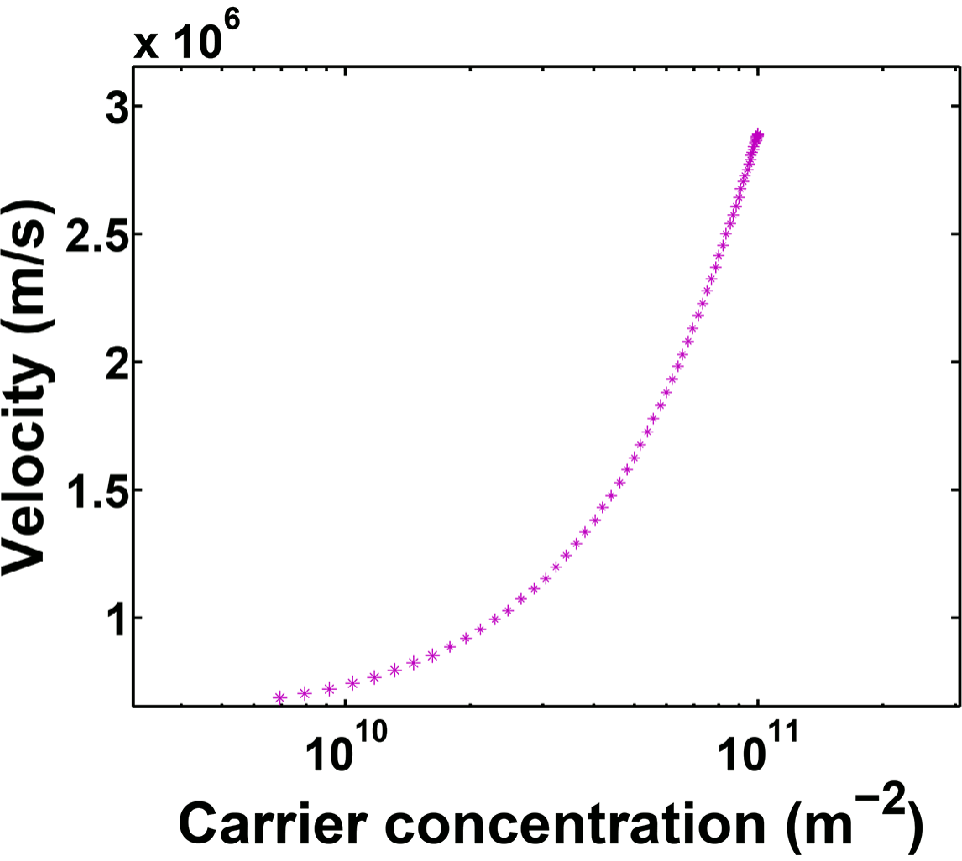
Carrier velocity versus carrier concentration in armchair graphene nanoribbon (AGNR).

Equation 11 can be solved numerically by applying the partial integration method [30]. The carrier velocity in bare AGNR versus the carrier concentration is plotted in Figure 2. Before exposure to the gas molecule (*t′* = 0), the value of the charge carrier density for the AGNR is *n* = 10^11^ cm^−2^, which is consistent with previous studies [31]. In addition, by increasing the number of carriers, the velocity of the electrons increases; thus, decreasing the carrier concentration will decrease the velocity.

To investigate the molecular adsorption effect on the carrier velocity, the current–voltage properties of the AGNR sensor based on the velocity relation with *I–V* can be derived as:

[12]
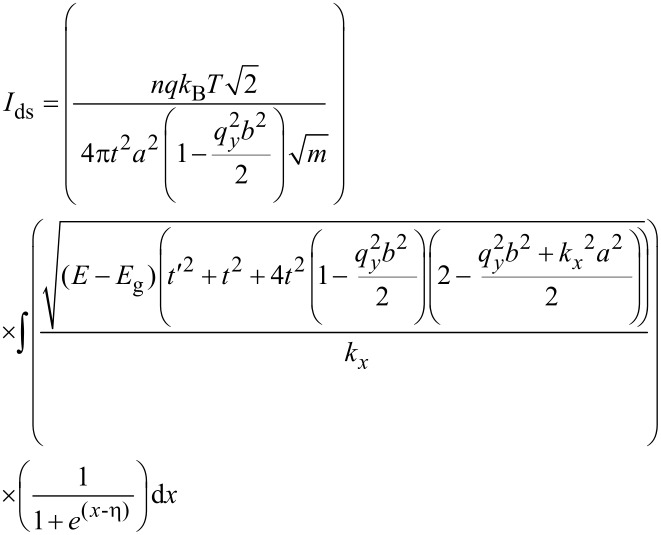


where *n* is the charge carrier density, and *q* is the electrical charge.

Besides the modelling study, the first principle calculation using the Atomistix Toolkit (ATK) is performed, which is based on density functional theory (DFT) and non-equilibrium Green's function formalism.

**Figure 3:**
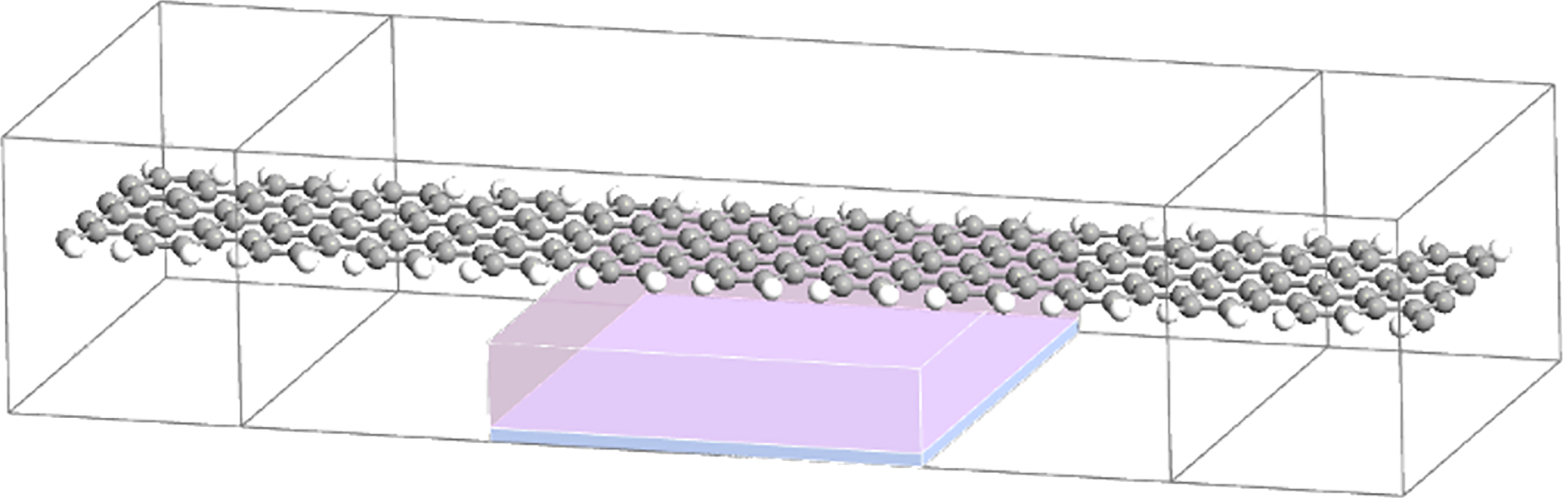
Illustration of the gas sensor with the 8-AGNR FET platform. The structure consists of the left and right electrode and the channel region in the middle with a metal back gate.

The physical properties of the GNRs are dependent on the width and edge shape of the ribbons. Figure 3 illustrates the schematic of a field effect transistor using an 8 atom-width ribbon referred to as 8-AGNR. This platform is used as a sensor platform.

To apply the sensor for gas detection, NO and CO adsorption on the AGNR surface are considered separately. Each gas molecule prefers a specific geometry when adsorbed on the AGNR plane. The AGNR-FET-based gas sensor is exposed to each of these gas molecules and its band structure and *I*–*V* characteristic variations before and after gas adsorption are studied; also, the adsorption energy and charge transfer between gas molecules and the AGNR surface are calculated and discussed.

**Figure 4:**
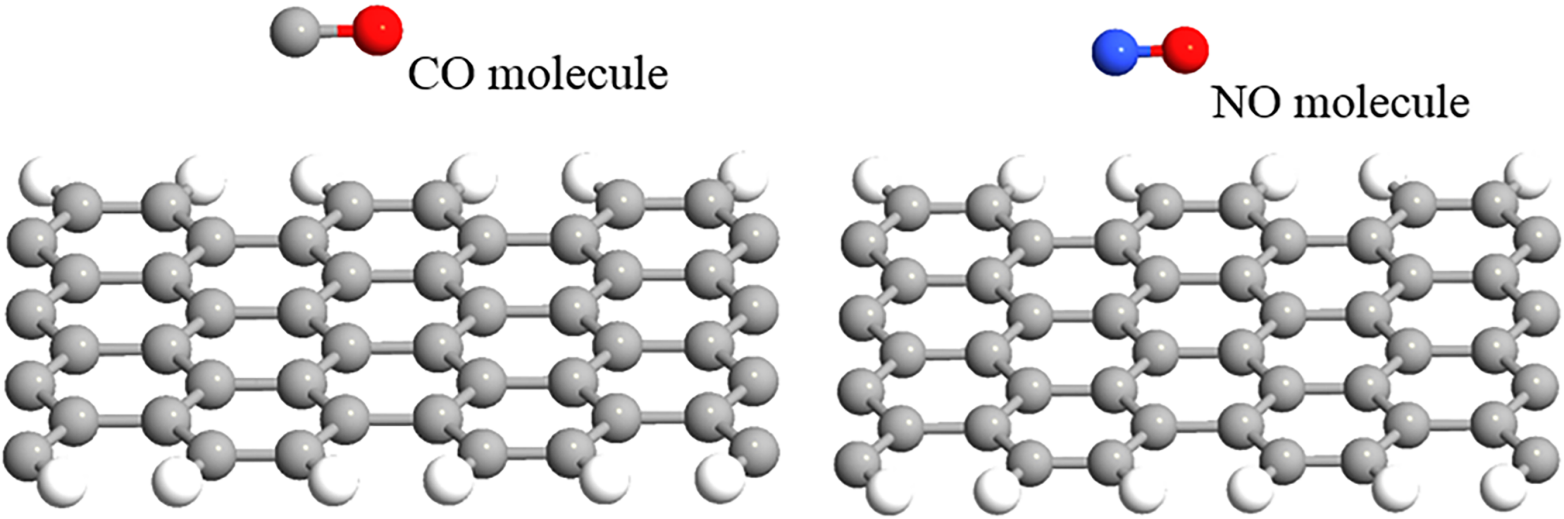
Orientation of CO and NO gas molecules on the 8-AGNR plane.

In the adsorption process of CO, different configurations were considered. Several orientations of the C–O bond above the AGNR plane were tested. After a complete relaxation of the structure, the optimal configuration for the CO molecule above the AGNR was found, where the C–O bond was parallel to the substrate and the molecular distance from the AGNR surface was 3 Å, as illustrated in Figure 4. In addition, several configurations of the NO molecule on the AGNR surface were considered for NO sensing. After molecule adsorption, the whole structure was optimized and relaxed so that the NO molecule was laid on the AGNR plane with a C–N distance of 3.74 Å and C–N–O angle of 90.1°. The bond length of N–O was 1.24 Å after adsorption, which was an increase by 0.07 Å, as presented in Figure 4.

The data presented in Table 1 gives the CO and NO adsorption energy on the AGNR and the amount of charge between each gas molecule and the substrate, which is calculated using Mulliken population analysis. The adsorption energy (*E*_a_) of the gas molecules on the AGNR is calculated by the expression that follows [32]:

[13]
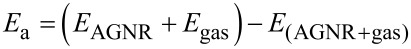


where *E*_(AGNR+gas)_, *E*_AGNR_ and *E*_gas_ are the total energy of the gas molecule on the AGNR, the relaxed AGNR, and a single gas molecule, respectively.

**Table 1:** Mulliken population and adsorption energy calculated for the adsorption of CO and NO molecules on the armchair graphene nanoribbon (AGNR).

Adsorbate	*E*_a_ (eV)	*Q* (eV)

NO	−2.1004	0.126
CO	−2.31724	−0.077

The results show a negative *E*_a_ for all gas molecules, which indicates an exothermic reaction between the AGNR and gas molecule according to Table 1. The magnitude of adsorption energy is larger than 1 eV for both NO and CO, indicating strong chemisorption.

On the other hand, referring to the Mulliken charge analysis [33], the charge transfer between the gas molecules and the AGNR is investigated. From the Mulliken charge analysis, the amount of the transferred charge between the AGNR and each gas can be calculated. In addition, we can determine whether the adsorbed molecule acts as an electron donating or withdrawing functional molecule. If the adsorbate is a donor, the value of the Mulliken charge must be positive; if it is negative, it is an acceptor molecule. According to Table 1, NO shows acceptor and CO shows donor properties [34–36].

**Figure 5:**
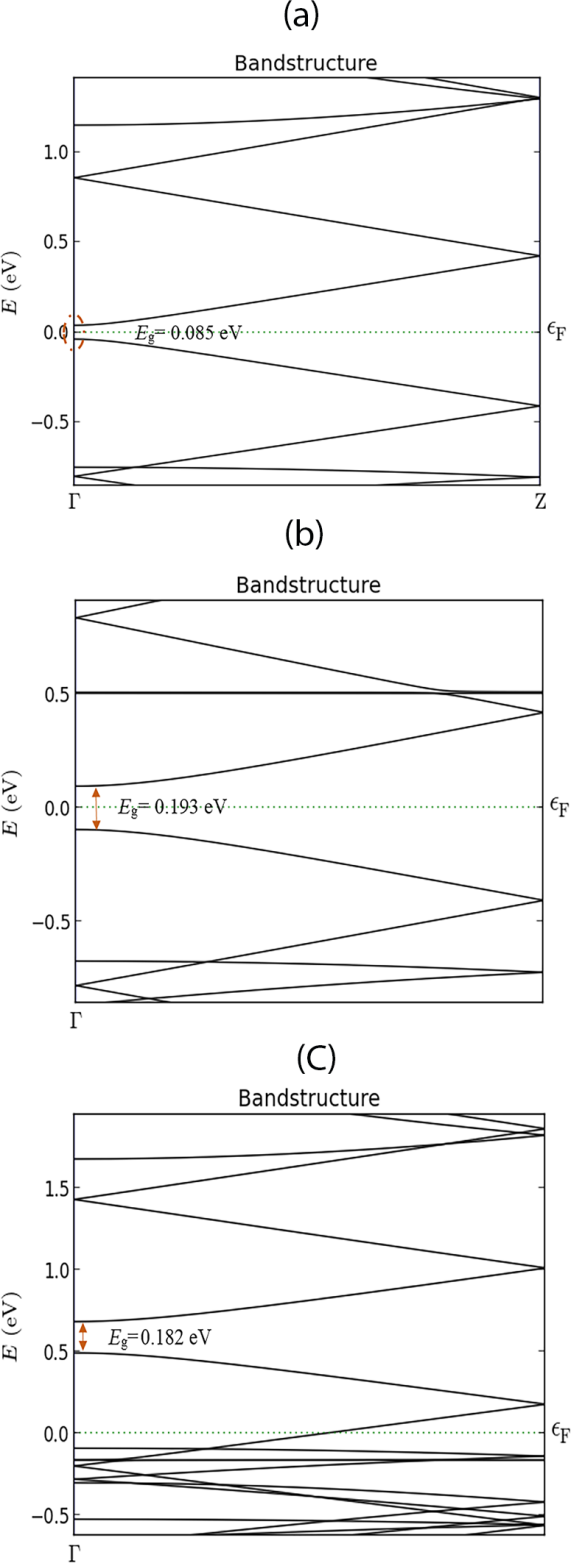
Energy band structure of the 8-AGNR: (a) bare AGNR, (b) after CO adsorption, (c) after NO adsorption.

The band structure analysis can further explain the gas interaction with the AGNR surface. The band structure of the AGNR before and after CO and NO adsorption are illustrated in Figure 5a–c.

The energy band diagram of the bare ribbon shows a very small band gap, *E*_g_ = 0.085 eV, that indicates semi-metallic properties. Furthermore, it is shown that the adsorption of gas molecules can modulate the AGNR band structure and modify the energy band gap. Based on Figure 5b, the band gap increased after CO adsorption on the AGNR. The conduction band level increased to *E*_C_ = 0.0952 eV above the Fermi level, and the valence band decreased to *E*_V_ = −0.0975 eV below the Fermi level. Therefore, the band gap was calculated to be *E*_g_ = 0.193 eV which shows a significant increase after gas adsorption. Furthermore, compared to the band structure of the clean system, the adsorption of NO has changed the band structure so that both the conductance and valence bands are shifted to above the Fermi energy level. The valence and conductance band energies were extracted to be *E*_V_ = 0.493 eV and *E*_C_ = 0.675 eV, respectively. Therefore, the energy band gap is *E*_g_ = 0.182 eV for a doped system. The band structure analysis shows that the Fermi level is located inside the valence band, indicating that NO can change the AGNR to a p-type semiconducting material.

For CO adsorption, the Fermi level was located inside the energy band gap, but slightly closer to the conduction band, which means that CO adsorption can lead to n-type semiconducting properties. After CO adsorption, an increase in the band gap and charge transfer occurred, which implies that CO acts as a donor; therefore, the CO adsorption can increase the electron concentration. Increasing the carrier concentration can increase the conductivity.

**Figure 6:**
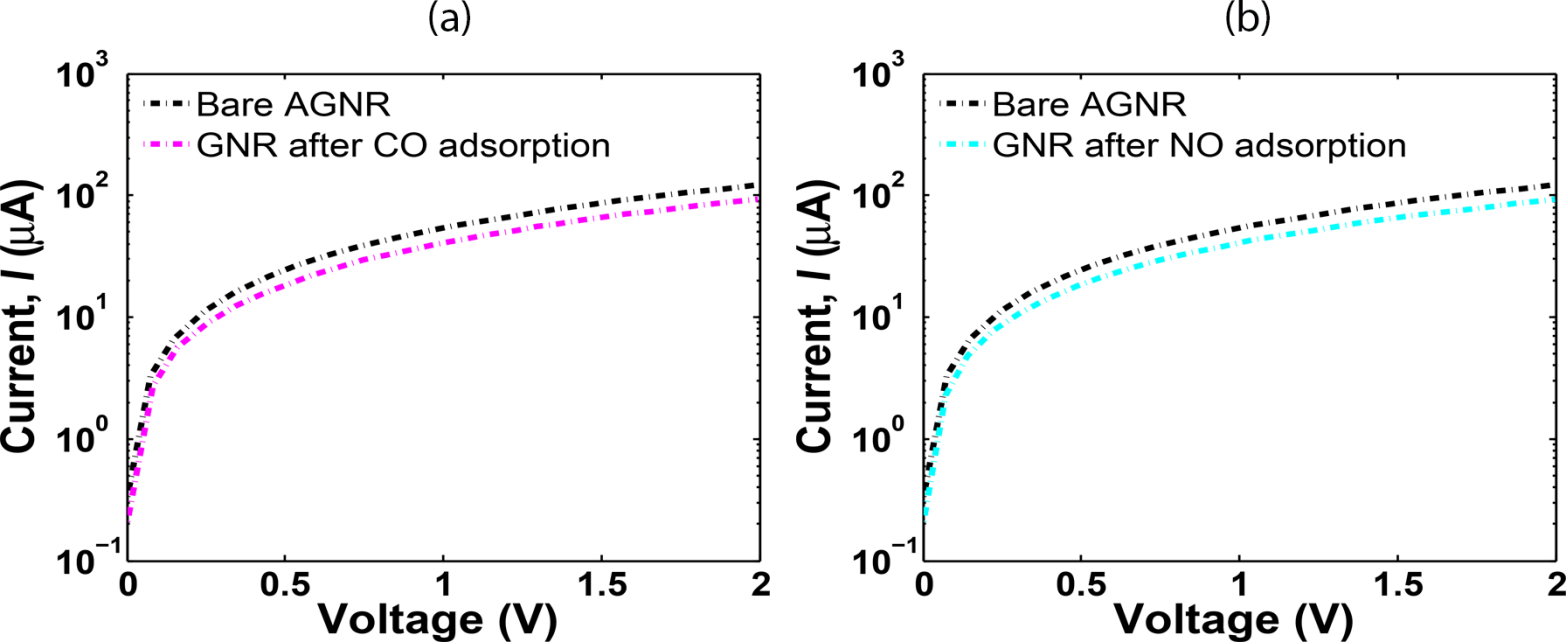
Armchair graphene nanoribbon (AGNR) sensor *I*–*V* characteristics obtained using the Atomistix Toolkit (ATK): (a) after CO and (b) after NO.

For NO adsorption, a reduction in the carrier concentration will decrease the AGNR conductivity. Furthermore, the energy gap increase results in a conductance decrease of the AGNR as well. These effects on the physical properties and the charge travel between the channel and gas molecules lead to some changes in the electrical characteristics of the AGNR-FET sensor. Figure 6a,b present the *I*–*V* analysis of the AGNR-FET before and after CO and NO adsorption.

It can be seen that the current has decreased after CO and NO adsorption because of the significant increase in the band gap. The *I*–*V* simulation results by ATK are compared with modelling results to demonstrate the functionality and correctness of our models. Based on the proposed carrier velocity model, the *I*–*V* relation of the sensor was formulated in Equation 12. To analyse the *I*–*V* characteristics of the sensor, the value of *t′* for each gas should be determined. The *t′* shows the coupling between the AGNR surface and the adsorbed molecule. The value of *t′* for each gas is different and can be determined based on the band structure analysis of AGNR after adsorption of each gas molecule. The *E*–*K* relation for the AGNR was formulated in Equation 6. By tuning the *t′*, we can adjust the band gap to obtain the calculated band gap that was presented before in Figure 5. The hopping integral parameters for NO and CO are extracted and presented in Table 2.

**Table 2:** The extracted hopping integral parameters for CO and NO adsorption, where *t′*_C–CO_ and *t′*_C–NO_ represent the hopping integrals between CO and NO gas molecules and the armchair graphene nanoribbon (AGNR) plane.

Hopping integral parameter	Band gap (eV)	Value (eV)

*t*′_C–CO_	0.193	4.37
*t*′_C–NO_	0.182	4.21

**Figure 7:**
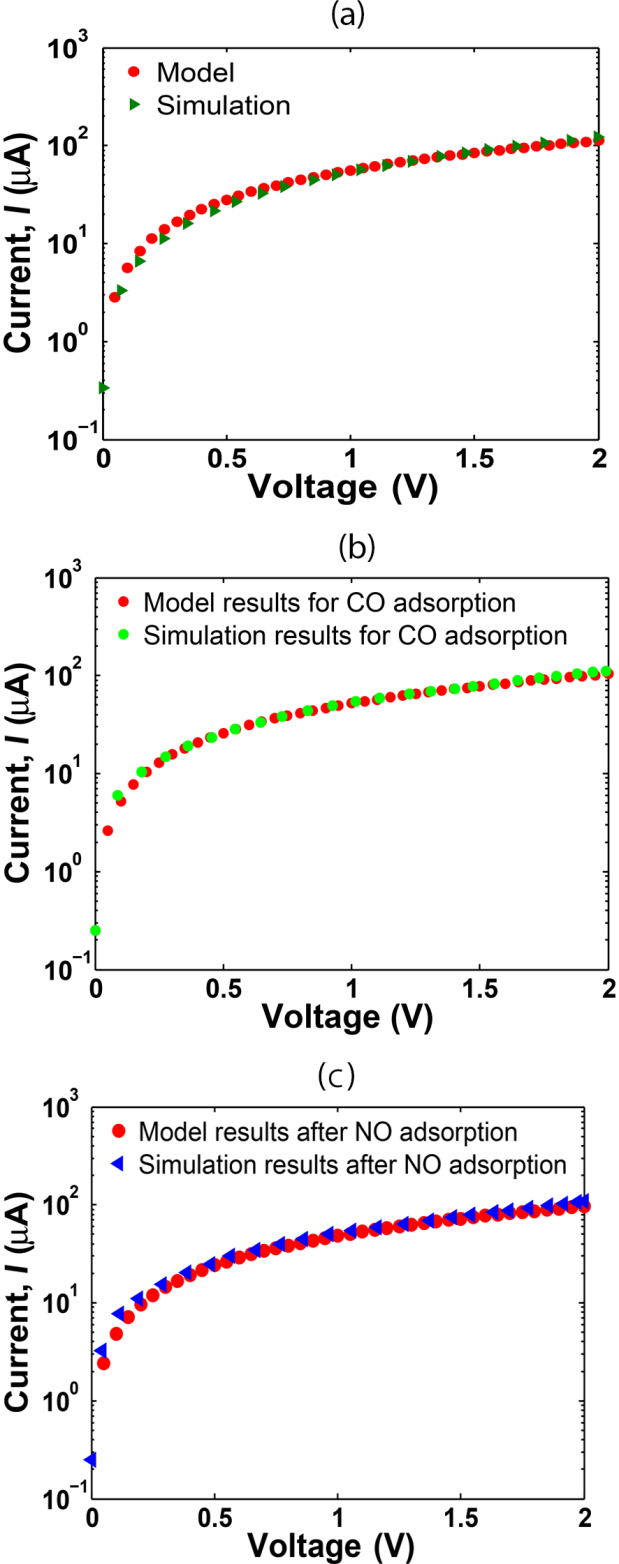
The comparison study for the *I*–*V* characteristics of the sensor simulated by MATLAB and the Atomistix Toolkit (ATK): (a) bare system, (b) after CO adsorption, and (c) after NO adsorption.

Based on Equation 12, the current–voltage properties of the AGNR sensor are illustrated in Figure 7a–c and the effects of the molecular adsorption are discussed.

For the bare AGNR, the value of *t′* is set to 0 eV. According to Figure 7a, the proposed model and simulation results show a perfect match for the clean system. In addition, the *I*–*V* comparison of the model and simulation data for CO and NO adsorption are presented in Figure 7b,c. A good consensus between our model and the ATK simulation results after CO and NO adsorption can be seen. It was expected that the current would decrease after NO adsorption and increase after CO adsorption, as CO is an electron donor while NO is an electron trapper. However, the current was shown to decrease after both NO and CO adsorption. This is because of the increased band gap of the AGNR after CO in comparison to NO adsorption, which implies a further decrease of the conductance after CO binding.

On the other hand, there are small differences between the model and simulation in some parts of Figure 7. This is because the proposed model is based on a perfect AGNR, while in the simulation by ATK, molecular forces among the gas molecules and the carbon atoms can lead to some modification of the AGNR structure and change in the C–C bond length of the AGNR that induces some discrepancy between the simulation and modelling results.

**Figure 8:**
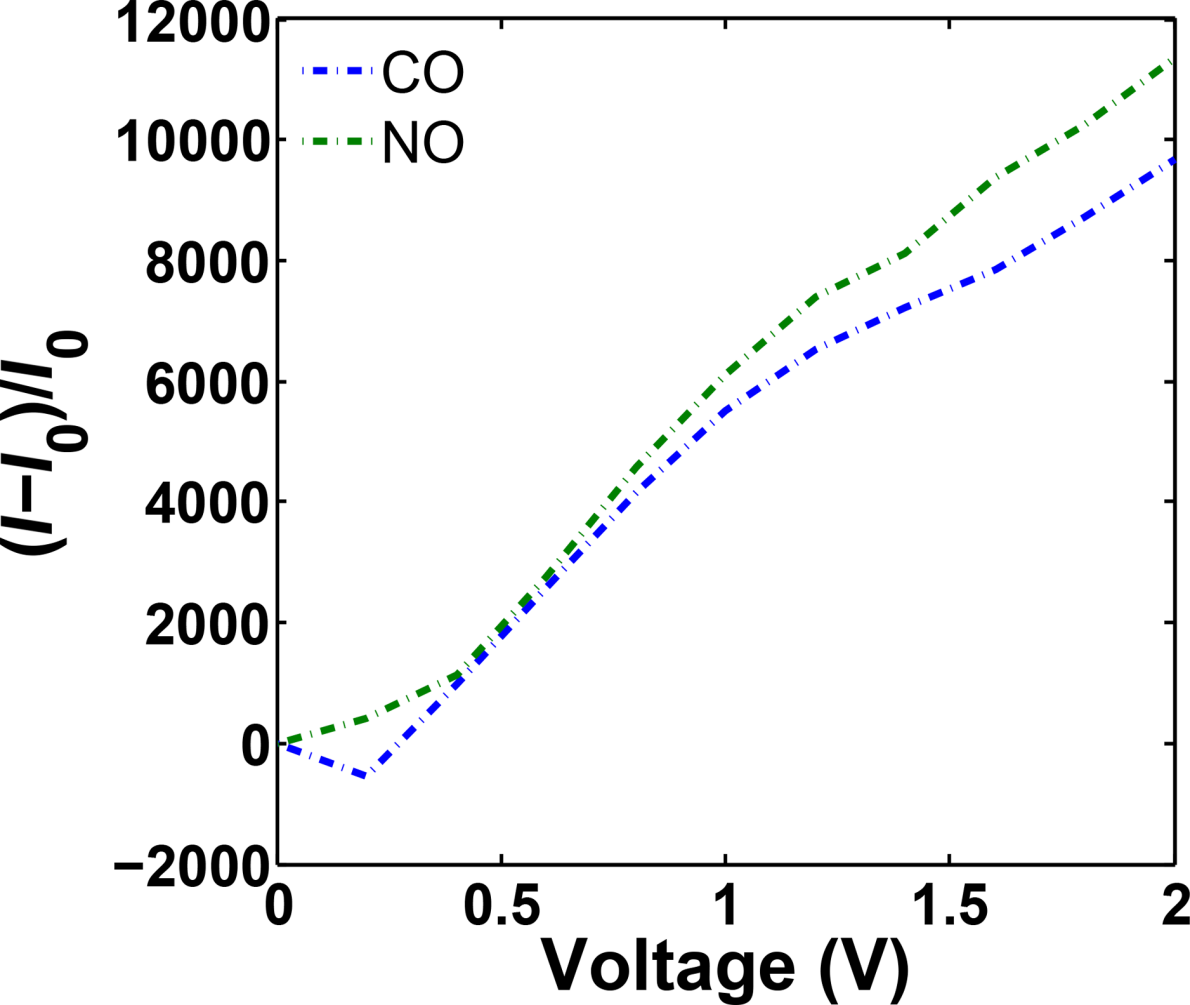
Sensitivity of the armchair graphene nanoribbon (AGNR) in the presence of CO and NO molecules.

The sensitivity of the sensor is also calculated for both NO and CO adsorption. Based on Figure 8, the sensitivity increases as the voltage increases and the sensor shows higher sensitivity toward NO adsorption. This demonstrates that AGNR is very sensitive toward its environment and is able to detect different gas molecules.

## Conclusion

In this research, molecular adsorption effects on the electrical properties of a AGNR-FET-based gas sensor were analytically modelled and investigated. To model the carrier velocity, the band structure, carrier concentration, and DOS of the AGNRs, including the coupling between the adsorbate and AGNR by introducing the hopping integral parameter (*t′*), was formulated. Then, the molecular adsorption effect on the carrier velocity was investigated in the form of current–voltage properties. The DFT calculations study was performed to further investigate gas molecule interactions with the AGNR surface and their effects on its physical and electrical properties. The band structure analysis indicated that the act of molecular adsorption could significantly modulate the AGNR band structure and modify its energy band gap. The increase of energy gap after adsorption of gaseous molecules results in the decrease of the conductivity of the channel that led to the reduction of the current of the sensor. Additionally, the Mulliken charge analysis confirmed that CO and NO molecules have an electron donor and electron acceptor nature, respectively. The comparison simulation study between the proposed model and first principles method showed an acceptable agreement between the results. Therefore, the proposed models in this research can be used to develop modern sensors based on the new materials.
